# Why Did the Bee Eat the Chicken? Symbiont Gain, Loss, and Retention in the Vulture Bee Microbiome

**DOI:** 10.1128/mBio.02317-21

**Published:** 2021-11-23

**Authors:** Laura L. Figueroa, Jessica J. Maccaro, Erin Krichilsky, Douglas Yanega, Quinn S. McFrederick

**Affiliations:** a Department of Entomology, Cornell University, Ithaca, New York, USA; b Department of Environmental Conservation, University of Massachusetts Amherst, Amherst, Massachusetts, USA; c Department of Entomology, University of California Riverside, Riverside, California, USA; d Department of Ecology, Evolution, and the Environment, Columbia University, New York, New York, USA; e Division of Invertebrate Zoology, American Museum of Natural History, New York, New York, USA; Harvard University

**Keywords:** corbiculate apid core microbiome, carrion, necrophagy, diet switch, pollinator ecology

## Abstract

Diet and gut microbiomes are intricately linked on both short and long timescales. Changes in diet can alter the microbiome, while microbes in turn allow hosts to access novel diets. Bees are wasps that switched to a vegetarian lifestyle, and the vast majority of bees feed on pollen and nectar. Some stingless bee species, however, also collect carrion, and a few have fully reverted to a necrophagous lifestyle, relying on carrion for protein and forgoing flower visitation altogether. These “vulture” bees belong to the corbiculate apid clade, which is known for its ancient association with a small group of core microbiome phylotypes. Here, we investigate the vulture bee microbiome, along with closely related facultatively necrophagous and obligately pollinivorous species, to understand how these diets interact with microbiome structure. Via deep sequencing of the 16S rRNA gene and subsequent community analyses, we find that vulture bees have lost some core microbes, retained others, and entered into novel associations with acidophilic microbes found in the environment and on carrion. The abundance of acidophilic bacteria suggests that an acidic gut is important for vulture bee nutrition and health, as has been found in other carrion-feeding animals. Facultatively necrophagous bees have more variable microbiomes than strictly pollinivorous bees, suggesting that bee diet may interact with microbiomes on both short and long timescales. Further study of vulture bees promises to provide rich insights into the role of the microbiome in extreme diet switches.

## INTRODUCTION

Diet can drastically influence the composition of a host’s gut microbiome. These effects manifest on both short and long timescales. In hosts with flexible diets, such as humans, diet can alter the gut microbiome over just a few days (1, 2). Over time, gut microbes can help hosts exploit novel food sources. For example, herbivorous, arboreal ant species harbor a greater abundance of bacteria than omnivorous, ground-dwelling ants, highlighting the importance of bacterial nitrogen recycling for hosts that consume diets poor in amino acids (3). Similarly, honey bees harbor bacteria that help them digest toxic sugars found in the nectar of certain plant species (4). The interplay between the gut microbiome and host diet can drastically impact host fitness in many animal species around the world, both vertebrate and invertebrate alike.

Diet shifts on long timescales are especially important across the insect order Hymenoptera. While the most recent common ancestor of wasps was likely phytophagous (5), pollen feeding in Anthophila (the bees) evolved more recently, likely from a predatory ancestor whose prey fed on pollen and nectar (6). Therefore, bees can be considered vegetarian wasps. In nature there are often exceptions, however, and some meliponine (stingless) bees have reverted from their vegetarian origins and now rely on carrion as their primary source of dietary protein (7). Some meliponines are facultatively necrophagous (8), meaning that they will consume fresh animal carcasses when available but will also forage for pollen and nectar. There are even records of two bumble bee species, Bombus terrestris (Linnaeus 1758) and Bombus ephippiatus (Say 1837), feeding on carrion (9). However, obligate necrophagy has been found in only three closely related *Trigona* species in the neotropics: T. hypogea (Silvestri 1902), T. necrophaga (Camargo and Roubik 1991), and T. crassipes (Fabricius 1793) (10). The obligate necrophages *T. hypogea* and *T. necrophaga* appear to completely eschew flowers, instead obtaining carbohydrates from extrafloral nectaries and fruits and protein from vertebrate carcasses (11, 12). There are two competing explanations for how the obligate necrophages use carrion. Noll et al. reported that *T. hypogea* chews flesh from the carcass, transports the flesh back to the colony in its crop, and deposits the flesh in wax pots where it is then mixed with honey; this mixture of honey and flesh then matures over a 14-day period into a paste rich in free amino acids and sugars (11). Roubik, Buchmann, and coworkers hypothesized that young workers use the consumed flesh to produce hypopharyngeal gland secretions, much like honey bees (13). In the latter scenario, it is the hypopharyngeal gland secretion that the colony stores in pots. Regardless of how they consume flesh, the obligately necrophagous stingless bees have abandoned their ancestral mutualism with flowering plants and earned the moniker “vulture bees” (14).

The role of microbes in the vulture bees’ extreme diet switch from pollinivory to necrophagy is a long-standing question. Early culturing studies found only several *Bacillus* spp. in the stored food of *T. hypogea* (13). More recent studies of stingless bees and other corbiculates suggest that the adult gut microbiota is highly conserved (15). The common ancestor to the corbiculate apids is thought to have associated with five distinct bacteria, and while there have been losses and gains of gut microbiome members across the corbiculate apid phylogeny, most species retain the original five (15). The role of these bacteria in host health has been most extensively studied in honey bees and bumble bees, where the microbiome has been found to play roles in nutrition, parasite defense, and detoxification (4, 16–19). Gut microbiomes of bees in the genus *Melipona*, however, lack the bacteria *Snodgrassella* and *Gilliamella*, which are ubiquitous in other corbiculate apids (20). Loss of these core symbionts in *Melipona* and greater variation in the stingless bee microbiome compared to the honey bee and bumble bee microbiomes may be explained by either ecological shifts or symbiont replacement (20). The gut microbiome of vulture bees is even more intriguing when viewed through the lens of an ancestral core microbiome, ecological shifts, and symbiont replacement. This is further compounded by the historic undersampling of vulture bees.

To build an understanding of whether the extreme diet shift of vulture bees led to symbiont replacement or whether the core gut microbiome adapted to this new diet, we here compare the gut microbiomes of pollinivorous, facultatively necrophagous, and obligately necrophagous stingless bees. By comparing microbiome compositions in closely related bees with differing diets, we aim to understand how diet shifts affect microbes that share a long evolutionary history with their hosts. We hypothesize two competing scenarios that are not mutually exclusive: (i) the diet shift may have led to symbiont extinction and replacement by microbes that can break down carrion, or (ii) the core stingless bee microbiome may persist, suggesting that these microbes evolved along with the bee over its diet shift and are adapted to a new protein source.

## RESULTS

**FIG 1:**
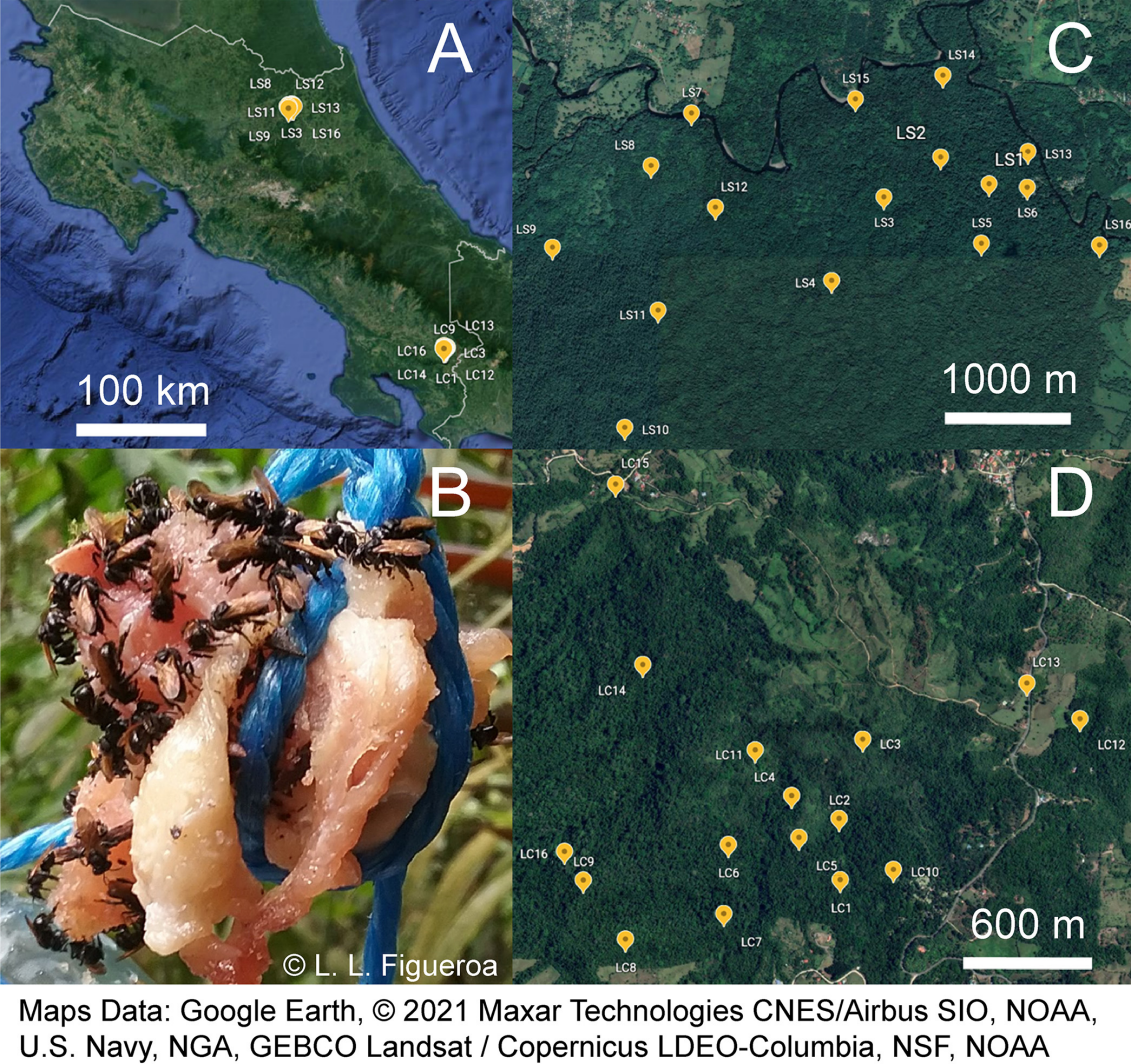
Locations and sampling design. (A) Map of Costa Rica and the two field stations where the 32 bait stations were deployed. (B) Example of a bait station with *Trigona* bees. (C) Bait stations deployed in La Selva Biological Station. (D) Bait stations deployed in Las Cruces Biological Station.

We collected 159 pollinivorous, facultatively necrophagous, and obligately necrophagous meliponine bees from 9 genera and 17 species from the La Selva and Las Cruces field stations in Costa Rica (Fig. 1 and see Table S1 in the supplemental material). On carrion baits, we collected 9 meliponine species from 4 genera (Table S2). We collected three species on chicken baits that, to our knowledge, are novel records for facultative necrophagy: Melipona costaricensis (Cockerell 1920), Partamona musarum (Cockerell 1917), and Partamona orizabaensis (Strand 1919). Other species of the same genera have been previously recorded from carrion baits (21). In agreement with Dorian and Bonoan (22), we also observed Trigona fulviventris (Guérin-Méneville 1844) and Trigona ferricauda (Cockerell 1917) carrying carrion in their corbiculae. We further observed carrion in the corbiculae of *P. musarum*.

10.1128/mBio.02317-21.1 TABLE S1 Additional information on sampling locations (location and date deployed). Download Table S1, XLSX file, 0.01 MB. Copyright © 2021 Figueroa et al. 2021 Figueroa et al. https://creativecommons.org/licenses/by/4.0/ This content is distributed under the terms of the Creative Commons Attribution 4.0 International license.

10.1128/mBio.02317-21.2 TABLE S2 Number of individuals from each species sampled and their corresponding dietary lifestyle (absent, facultative, or obligate necrophagy). Download Table S2, XLSX file, 0.01 MB. Copyright © 2021 Figueroa et al. 2021 Figueroa et al. https://creativecommons.org/licenses/by/4.0/ This content is distributed under the terms of the Creative Commons Attribution 4.0 International license.

We obtained a total of 12,972,564 16S rRNA gene amplicon reads with an average of 73,707 reads per sample. After quality control, chimera removal, decontamination, and removal of chloroplast and mitochondrial reads, we retained an average of 14,151 reads per sample across 172 samples. Across all samples, we identified a total of 1,937 amplicon sequence variants (ASVs).

**FIG 2:**
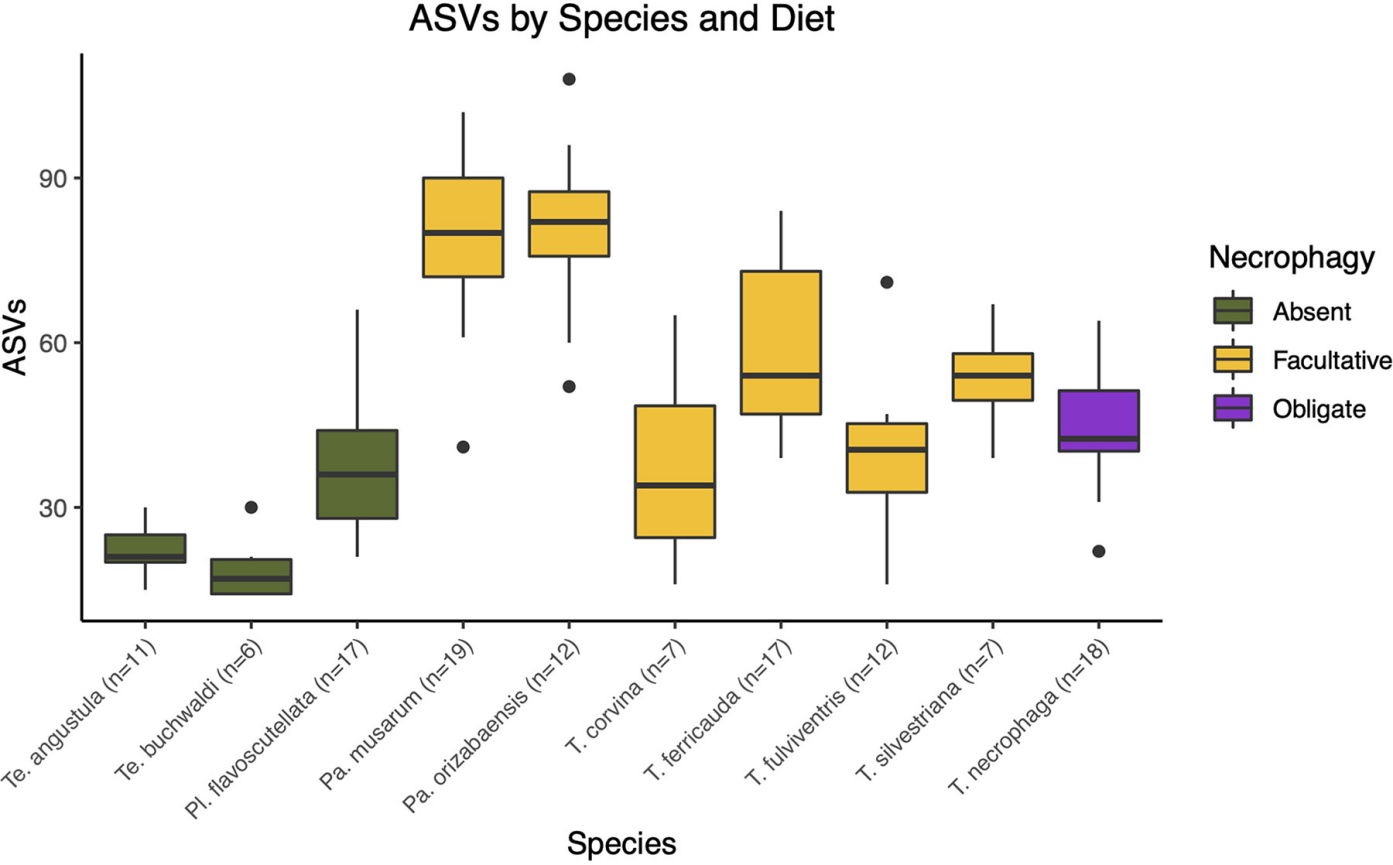
Number of observed ASVs by species and diet. Pollinivores had the lowest ASV richness while facultative necrophages had the highest ASV richness.

Species was a highly significant predictor of ASV alpha diversity in the bees (χ^2^_9_ = 125.34, *P* < 0.001), while diet was only marginally significant after controlling for species (χ^2^_2_ = 5.98, *P* = 0.050). Conversely, collection substrate was not a significant predictor of ASV alpha diversity (χ^2^_2_ = 4.52, *P* = 0.104), likely because facultative species were found across the collection substrates, further highlighting the importance of species and diet. Pairwise comparisons among species indicate several significant differences in ASV alpha diversity (Table S3). Two *Partamona* species harbored the greatest number of ASVs while two *Tetragonisca* species harbored the fewest ASVs (Fig. 2).

Across all samples, the most abundant ASVs were assigned to the genus *Snodgrassella* (Table S4). The other corbiculate apid “core” bacteria (*Bombilactobacillus* “Firm 4” and *Lactobacillus* “Firm 5,” *Gilliamella*, and *Bifidobacterium*) were also present but not as abundant as *Snodgrassella.* Other noncore bacteria that are nevertheless commonly associated with stingless bees were also abundant in some samples: *Bombella*, *Commensalibacter*, and an *Acetobacter-*like bacterium (15). *Melipona* harbored lactobacilli, *Bifidobacterium*, and *Convivina* (Table S4). Environmental bacteria, including lactobacilli that associate with flowers, such as Apilactobacillus ozensis, were also present at high relative abundance in some samples.

10.1128/mBio.02317-21.3 TABLE S3 Comparisons in amplicon sequence variants (ASVs) between bee species. Bold indicates *P* < 0.05. Download Table S3, XLSX file, 0.01 MB. Copyright © 2021 Figueroa et al. 2021 Figueroa et al. https://creativecommons.org/licenses/by/4.0/ This content is distributed under the terms of the Creative Commons Attribution 4.0 International license.

10.1128/mBio.02317-21.4 TABLE S4 Feature table with raw amplicon sequence variant (ASV) counts and taxonomy. We used two methods of classification to assign taxonomy to each ASV: naïve Bayesian classification and BLASTn. TopBLASTID, TopBLASTAccNo, and PercentID are output from BLASTn, while we obtained NaiveBayesClassification and Conf from the QIIME2 sklearn classifier. Count is the number of times each ASV was observed across all samples, while prevalence corresponds to the percentage of each ASV relative to the total count of all ASVs observed across all samples. Beginning in column J until column FY, each column represents a sample. Below the dietary lifestyle indicator (Absent, Facultative, and Obligate necrophagy) is the total number of ASVs for that sample, followed by the species name and the sample ID. Then, for each sample, the value in every subsequent row indicates the number of times that each ASV was found in that given sample. Wasps and bait (chicken) sample are also included. Download Table S4, XLSX file, 1.1 MB. Copyright © 2021 Figueroa et al. 2021 Figueroa et al. https://creativecommons.org/licenses/by/4.0/ This content is distributed under the terms of the Creative Commons Attribution 4.0 International license.

**FIG 3:**
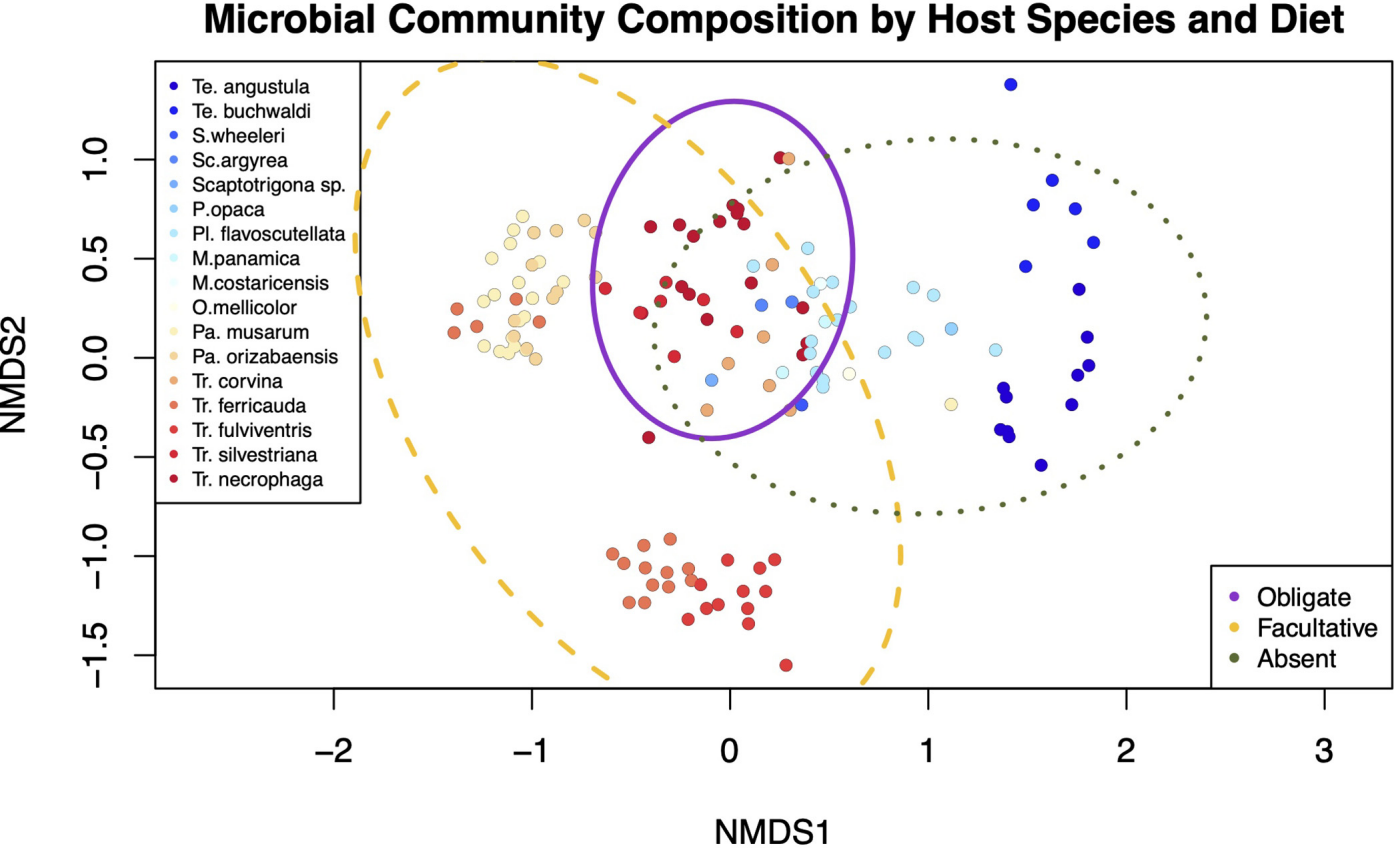
Nonmetric multidimensional scaling (NMDS) ordination of gut microbial communities by host species and diet. Since we found significant clustering by both species and diet, we represent species by the color of the points and demarcate diet by the ellipses (95% confidence intervals). Species NMDS stress = 0.08 and diet NMDS stress = 0.09 (*k* = 5 for both).

The composition of bee gut microbiomes and beta dispersion also differed by diet (Fig. 3, adonis F = 9.20, df = 2, *P* = 0.001, and betadisper F = 11.97, df = 2, *P* = 0.001). Pairwise comparisons in beta dispersion were largely significant, with the exception of absent and both facultative and obligate necrophagous diets (Table S5). Gut microbiome composition also differed at the species level (Fig. 3, adonis F = 2.25, df = 9, *P* < 0.001), while beta dispersion did not significantly differ between species (F = 0.96, df = 9, *P* = 0.740).

10.1128/mBio.02317-21.5 TABLE S5 Matrix displaying the *P* values from permuted pairwise comparisons of beta dispersion among different diets (absent, facultative, and obligate necrophagy). Observed *P* value below the diagonal and permuted *P* value above the diagonal. Download Table S5, XLSX file, 0.01 MB. Copyright © 2021 Figueroa et al. 2021 Figueroa et al. https://creativecommons.org/licenses/by/4.0/ This content is distributed under the terms of the Creative Commons Attribution 4.0 International license.

**FIG 4:**
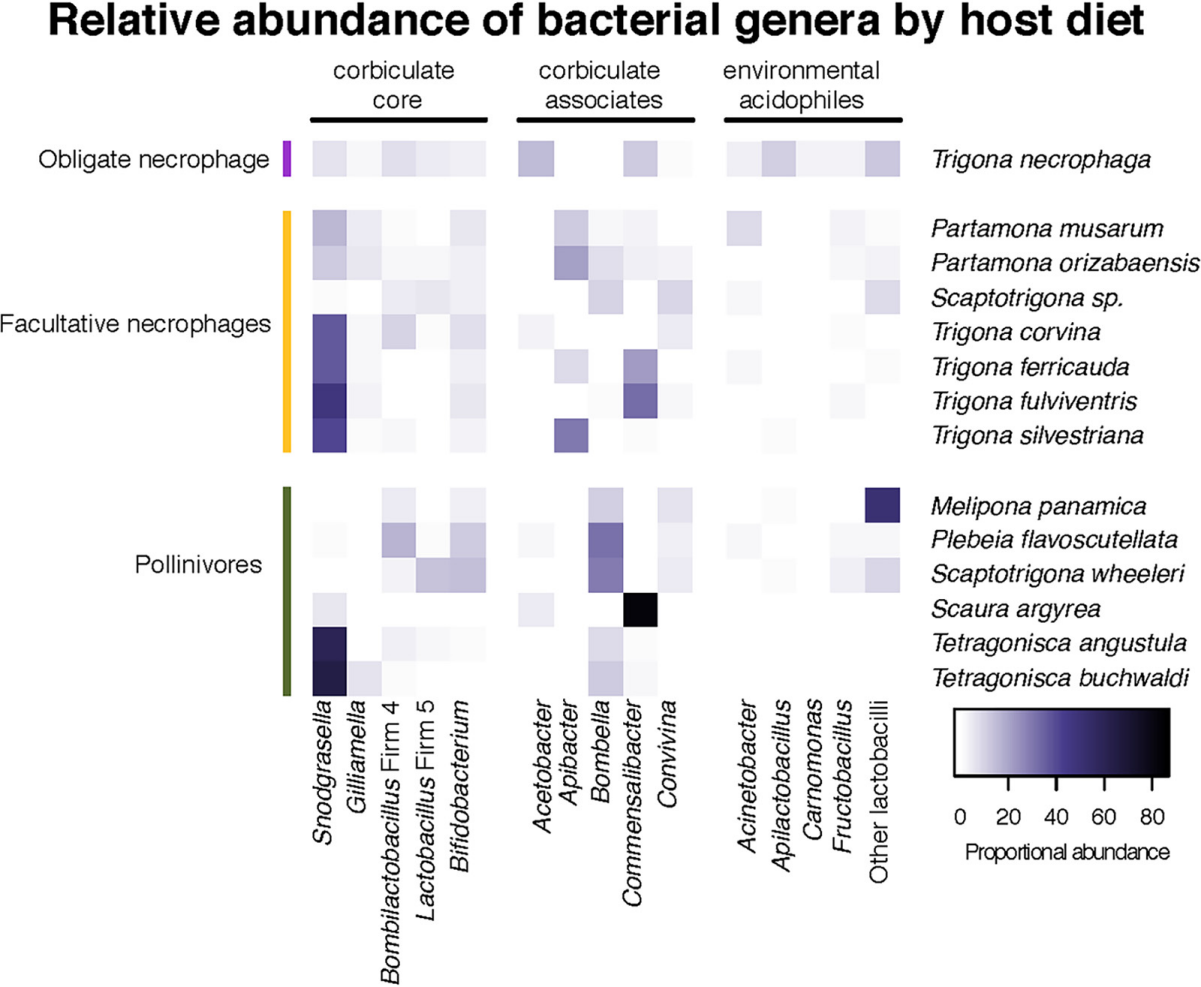
Average relative abundance of corbiculate core, corbiculate associates, and environmental bacteria by host species and diet. Species from which a single specimen was captured (*M. costaricensis*, Oxytrigona mellicolor, and Paratrigona opaca) are not included. Acidophilic microbes that have been found in the environment or on meat (e.g., *Apilactobacillus* and *Carnomonas*) were significantly more abundant in vulture bees while corbiculate core bacteria were significantly more abundant in obligate pollinivores (e.g., *Bifidobacterium* and *Snodgrassella*).

The obligate necrophages harbored 32 ASVs at greater relative abundances than pollinivores or facultative necrophages (Fig. 4 and Table S6). These differentially abundant ASVs included a lactic acid bacterium (LAB) whose top BLAST hit was to Apilactobacillus kosoi (97.6% identity) that was present in 95% of the *T. necrophaga* samples. Other ASVs that were at greater abundance in the necrophagous species include LAB and LAB-like bacteria associated with meat: *Carnobacterium*, *Brochothrix*, and *Vagococcus* (23–25). *Carnobacterium* and *Vagococcus* were also both identified on the chicken baits used to capture bees and in the wasps that were caught on these chicken baits (Table S6). *Acetobacteraceae* (AAB) ASVs that had top BLAST hits to *Commensalibacter* and *Acetobacter* were also overrepresented in the obligate necrophages.

10.1128/mBio.02317-21.6 TABLE S6 Differential abundance of each amplicon sequence variant (ASV) by diet. Table includes taxonomy of ASV. Based on a null hypothesis of equal relative abundance of each ASV across groups, if the null is rejected (reject null column is TRUE), then there is a significant differential abundance in the specified diet. If FALSE, there is not a significant difference in the abundance of ASVs between diet types. The W value is a count of the number of subhypotheses that have passed for a given ASV. Rows colored by whether they are differentially abundant obligate necrophages (peach), facultative necrophages (purple), or pollinivores (aquamarine). The three final columns (100Absent, 100Facultative, and Necrophagy) indicate the number of sequences that were assigned to the ASV of that row across all samples of that diet. Download Table S6, XLSX file, 0.5 MB. Copyright © 2021 Figueroa et al. 2021 Figueroa et al. https://creativecommons.org/licenses/by/4.0/ This content is distributed under the terms of the Creative Commons Attribution 4.0 International license.

In the facultatively necrophagous species, Acinetobacter, *Bifidobacterium*, and *Enterococcus* ASVs were overrepresented (Fig. 4 and Table S6). Pollinivorous species harbored abundant corbiculate apid core microbes compared to facultative or obligate necrophages (Fig. 4 and Table S6). *Bifidobacterium*, *Snodgrassella*, and *Bombilactobacillus* “Firm 4” and *Lactobacillus* “Firm 5”—all members of the corbiculate core microbiome (15)—were significantly more abundant in pollinivores. AAB with top BLAST hits to *Bombella*, *Gluconobacter*, and *Neokomagataea* that either are commonly associated with bees (*Bombella*) or have been isolated from flowers (25–27) were also more abundant in pollinivorous bee guts.

## DISCUSSION

Reversion to a carnivorous lifestyle in the obligately necrophagous bee *Trigona necrophaga* involved retention of some ancestral core microbes as well as acquisition of new lactic acid bacteria (LAB) and acetic acid bacteria (AAB). The extreme diet switch from pollen to carrion was likely facilitated by or resulted in the novel composition of the vulture bee microbiome. This new microbiome is likely to provide novel functions to its host. For example, mammalian carnivore microbiomes are enriched in amino acid degradation pathways while mammalian herbivore microbiomes are enriched in amino acid synthesis pathways (28). Our findings suggest that the vulture bee microbiome has adapted to the host’s novel diet by a combination of novel symbiont recruitment, loss of some ancestral microbes, and possible adaptation of some ancestral microbes.

While carrion is an abundant source of nutrition, there are barriers that must be overcome for necrophagous animals (29). Burying beetles face competition with microbes that degrade the nutritional quality of carcasses and prefer fresh carcasses when possible (30). Carrion-decomposing microbes produce toxic compounds, likely as a way to reduce competition with vertebrate scavengers (31). Carcasses are also sources of pathogens originating from the host, some of which can survive passage through a vulture’s digestive tract (32). While specialized immunity and low gut pH appear to be important adaptations to the carrion-eating lifestyle, having a specialized microbiome has also been found to be important (33). For example, the gut microbiomes of alligators, black vultures, and turkey vultures are dominated by *Clostridia* and *Fusobacteria* (34). These bacteria are also found on the carcass itself and thrive in the harsh environment of the vulture gut, suggesting that these microbes are important for adaptation to carrion feeding in vultures (34). While *Fusobacteria* are also found in carrion beetle gut microbiomes, they are minor constituents, while a *Xanthomonadales* bacterium related to a flesh-eating fly-associated bacterium is abundant across eight species of burying beetles (35). Across necrophagous animals, it appears that unique microbes that aid in digestion are ubiquitous.

Like vultures and carrion beetles, vulture bees also harbor unique microbes that are associated with their carrion diet. Surprisingly, carrion-associated microbes did not dominate the vulture bee microbiome. Instead, acidophilic microbes that either were found in the environment or are known to associate with other corbiculate bees were more abundant in the vulture bee microbiome. *Gilliamella* and *Snodgrassella*, which are part of the ancestral corbiculate core (15), are present only in low abundance and prevalence in vulture bees. *Snodgrassella*, however, is abundant in the microbiomes of facultative and pollinivorous bees, suggesting that its importance in the microbiome of vulture bees has diminished. This is not unprecedented; the bee genus *Melipona* has recently been found to have lost both *Snodgrassella* and *Gilliamella* (20), which our sampling corroborates. On the other hand, *Lactobacillus* “Firm 4” and an *Acetobacter-*like bacterium are considered ancestral in stingless bees (15), and these bacteria are retained in vulture bees. The retention of these ancestral microbes suggests either that they have adapted to the carrion protein that the bees ingest or that they serve vital functions for the bee regardless of diet. Comparative genomics of these bacteria are needed to disentangle these two possibilities. Perhaps most surprisingly, vulture bees harbor abundant lactobacilli in the Apilactobacillus micheneri clade, a group of bacteria which we did not find on the baits but that are frequently found on flowers and in solitary bee pollen provisions and guts (36–38). Since *T. necrophaga* is not known to visit flowers, these bacteria may also occur in the extrafloral nectaries and fruit that necrophagous bees visit for carbohydrates; this hypothesis is supported by the isolation of *Apilactobacillus kosoi*, a later heterotypic synonym of *A. micheneri*, from a fermented vegetable drink (39, 40). However, we do not exclude the possibility that *T. necrophaga* acquires *A. micheneri* from naturally occurring carrion (not sampled here). Experimentally assessing the role of diet in the microbiome of both obligate and facultatively necrophagous bees would clarify whether the patterns found here represent a long-term association or are more malleable and driven by environmental acquisition. Similarly, comparative genomics of the *Apilactobacillus* strains isolated from vulture bees and pollinivorous solitary bees will be fascinating, given their cooccurrence in both groups despite markedly different dietary lifestyles.

LAB and AAB are important symbionts of insects, including bees (41–43). For example, lactobacilli that colonize bumble bee guts can inhibit the growth of a gut pathogen via lactic acid production (19). Honey bee guts are acidified by the presence of a gut microbiome, especially in the ileum and rectum where *Snodgrassella* (ileum) and lactobacilli (rectum) are dominant (16, 44). While we did not measure bee gut pH in our study, the dominance of acidophilic bacteria in the vulture bee gut suggests that an acidic gut environment is important for these bees as well. Gut acidification appears to be an important adaptation for necrophagous animals. For example, genes involved in vulture gastric acid secretion exhibit signatures of natural selection, and the acidic gut of vultures is thought to defend the birds against pathogens obtained in their food (33, 45). Similarly, gastric acids and enzymes work together to digest meat in the human gut (46), suggesting that meat digestion may be facilitated by acidophilic bacteria. These patterns have even been observed outside hosts, as LAB prevent growth of spoilage and pathogenic bacteria in preserved meats via acidification, bacteriocins, and H_2_O_2_ (47). These possible functions warrant further study in vulture bees.

The ASVs that were more abundant in facultatively necrophagous bees included both environmental and corbiculate core microbes. While *Bifidobacterium* is a member of the corbiculate core (15), some Acinetobacter bacteria are commonly found in floral nectaries and have been reported in the pollen provisions of small carpenter bees (48, 49). *Enterococcus* is a large genus of LAB that includes species that have been isolated from honey bee guts (50). The differential abundance of these environmental microbes in facultatively necrophagous bees suggests that flexible stingless bee diets lead to greater ASV variation, a finding that is also supported by the highest ASV richness in facultatively necrophagous bees. Diet therefore appears to interact with the microbiome of stingless bees on both short and long timescales. Interestingly, there is a parallel in mammals, where both herbivores and carnivores harbor specialized bacterial lineages, unlike omnivores, which do not have specialist bacteria and instead harbor a combination of bacterial groups from both herbivores and carnivores (51). It is important to note that while a change in diet could have modified the microbiome, it is also possible that a shift in microbiome enabled a change in dietary lifestyle, or even that both the change in microbiome and the change in diet were linked to a different unmeasured phenomenon in the evolutionary history of these unique bees. Assessment of the functional role of these microbes in facultative and obligate necrophagous bees is necessary to disentangle these possible hypotheses.

The microbiome composition of pollinivorous stingless bees in our study largely agrees with previous findings (15). The corbiculate core is present in the pollinivorous stingless bees, albeit with greater variation than that seen in honey bees and bumble bees. The bacteria that were more abundant in pollinivorous stingless bees were mostly corbiculate core ASVs like *Bifidobacterium*, *Snodgrassella*, *Bombilactobacillus* “Firm 4,” and *Lactobacillus* “Firm 5.” These results suggest that strict pollinivory may help maintain associations with corbiculate core microbes. Changes in gut morphology between these bees could explain some of the differentially expressed ASVs. For example, given that *Snodgrassella* is an ileum colonizer (44), pollinivorous species may have higher relative abundance than necrophagous bees if the shift in dietary lifestyle resulted in a reduction in ileum size, though this remains to be tested. Similarly, while in this study we focused on relative abundance, some taxa with high relative abundance in small communities may be comparable in absolute number to taxa with low relative abundance in larger communities.

Stingless bees, occurring in tropical and subtropical ecosystems around the world, have a unique combination of life history traits that enable diverse and largely understudied microbial symbioses (52). Tragically, deforestation rates continue to increase throughout much of Latin America, resulting in extensive losses to biodiversity (53), likely before species and microbial symbioses have been fully described. Costa Rica was the only country out of 15 evaluated in Latin America to have, on average, an increase in forest cover from 1980 to 2010 (54). Thus, our success in locating vulture bees was likely greater than it would have been elsewhere where there was less natural habitat, further highlighting the need to conserve these biodiversity hot spots for the immense vertebrate, invertebrate, and microbial diversity found there.

In summary, we found evidence that reversion to a carnivorous lifestyle in an obligate necrophagous bee had profound consequences for its microbiome. The enrichment of acidophilic bacteria in vulture bee guts necessitates future functional studies. We recommend shotgun metagenomic studies as the logical next step, followed by experimental manipulation to untangle putative function. Another fascinating line of study would be to look at the host range of stingless bee-associated microbes to better understand the roles of coevolution and partner choice and how that varies with diet. We propose that these fascinating bees will offer rich insights into how diet interacts with gut microbiomes.

## MATERIALS AND METHODS

### Field methods.

We collected adult bees at the Organization of Tropical Studies (OTS) La Selva and Las Cruces field stations in April of 2019 (Fig. 1). At each field station, we set up 16 bait stations consisting of approximately 50 g of fresh chicken suspended from branches with string. Each bait was hung approximately 1.5 m off the ground, and the string was coated with petroleum jelly to deter ants and other nonflying visitors, though occasionally bullet ants (Paraponera clavata) were able to overcome the barrier. We set up multiple bait stations per location to maximize the probability that we collected from multiple colonies; as meliponines use chemical cues to recruit colony mates to a food source (55), bees collected at a single bait may not be independent samples (Fig. 1B is an example of many workers likely from a single colony). The latitude and longitude coordinates and dates of initial bait placement can be found in Table S1 in the supplemental material. After initial placement, we visited the bait stations daily for the following 5 days and collected bees visiting the bait or patrolling the area. If a bee was flying near the bait but not caught on the bait, we labeled it as “patrolling,” while we labeled bees caught while collecting meat as “bait.” Furthermore, we opportunistically collected bees from flowers and from colony entrances, again recording the collection source. We used these collection records and published literature to bin bee species by necrophagy: “absent” for bees that we never observed on baits and were not recorded in the literature as collecting from carcasses, “facultative” for bees known from the literature to facultatively forage on carrion and those that we collected on baits but were not recognized in the literature as obligate necrophages, and lastly “obligate” for *Trigona necrophaga* (10). We identified species using an unpublished key to the stingless bees of La Selva (Paul Hanson, Escuela de Biología, Universidad de Costa Rica) and the La Selva Museum collections. In total, we collected 159 meliponine bees from 9 genera and 17 species (*n* = 1 to 26 per species, average *n* = 9.35 per species, Table S2). For comparison to the meliponine samples, we additionally collected 8 wasps that were foraging on the chicken baits and 5 chicken bait samples across 4 days. We worked under permit R-013-2021-OT-CONAGEBIO for the collections and subsequent molecular analyses.

### Sample processing.

We collected each bee into a sterile tube filled with 95% ethanol. We stored samples at room temperature in the field and during transport and at −80°C at the University of California, Riverside, until ready for DNA extraction. Due to the small size of the bees and the small amount of microbial biomass on insect exoskeletons (56), we used entire abdomens for all bees except for the larger *Melipona* bees (*n* = 4), for which we dissected out the gut under sterile conditions. We placed each sample into a tissue collection plate (Qiagen, Germantown, MD) and added two 3-mm chromium steel beads and ∼50 μl of 0.1-mm zirconia beads (Biospec, Bartlesville, OK). Next, we added 180 μl of Qiagen buffer ATL and 20 μl of proteinase K to each sample. To lyse recalcitrant cells, we bead beat the samples on a Qiagen Tissue Lyser for 6 min at 30 Hz, after which we rotated the plates and performed a second round of bead beating for 6 min at 30 Hz. We then incubated the samples at 56°C for an hour and then followed the Qiagen DNeasy Blood and Tissue protocol for the remainder of the extraction process. We included 3 blank extractions as no-template controls in all downstream procedures and analyses.

### 16S rRNA gene amplicon sequencing.

To characterize the bacterial community in each gut, we then prepared amplicon libraries using the 799F (CMGGATTAGATACCCKGG)-1115R (AGGGTTGCGCTCGTTG) 16S rRNA gene primers (57, 58). We have used this primer pair extensively to prepare and sequence dual inline barcoded libraries (48, 59–61). Briefly, we first included the partial Illumina sequencing adapter and a unique combination of 8-mer barcodes for each sample with primers that include the genomic primer sequence. We performed 25 cycles of PCR in 25-μl reaction mixtures with a 52°C annealing temperature using Phusion MasterMix (Thermo Scientific, Waltham, MA). To clean these reaction mixtures, we used exonuclease and shrimp alkaline phosphatase to remove excess primers and deoxynucleoside triphosphates (dNTPs), respectively. We then performed a second PCR with 1 μl of cleaned PCR product as the template and primers that complete the Illumina sequencing construct: PCR2F (CAAGCAGAAGACGGCATACGAGATCGGTCTCGGCATTCCTGC) and PCR2R (AATGATACGGCGACCACCGAGATCTACACTCTTTCCCTACACGACG). Both PCRs start with an initial 94°C denaturing step for 3 min followed by 25 cycles of 94°C for 45 s, 52°C for 1 min, and 72°C for 1-1/2 min. To normalize the amount of DNA in each library, we used SequalPrep normalization plates (Invitrogen, Waltham, MA), following the manufacturer’s protocol. We used 5 μl of each library to create a library pool, which we then cleaned with AMPure XP beads (Beckman Coulter, Brea, CA) to remove primer-dimers and excess master mix components. We checked the quality and concentration of the pooled libraries using the 2100 Bioanalyzer (Agilent. Santa Clara, CA). The Genomics Core at the University of California, Riverside, then sequenced the libraries on the MiSeq (Illumina, San Diego, CA) using the V3 2 × 300 reagent kit.

### Bioinformatics and statistics.

We used QIIME2 to process the Illumina fastq files and conduct initial analyses (62). Before demultiplexing the sequences, we first removed the barcodes and concatenated them into a separate barcode file so that the format was compatible with QIIME2. For quality control, chimera removal, and binning of reads into amplicon sequence variants (ASVs), we ran DADA2 with default parameters and read trimming of 20 bases for forward reads and 80 bases for reverse reads (63). We then assigned taxonomy to ASVs using two methods. First, we trained the QIIME2 sklearn classifier to the 799 to 1115 region of the SILVA 16S rRNA gene database (64, 65). Second, we used NCBI’s 16S rRNA database (accessed 24 June 2021) to conduct local BLAST searches and a custom perl script to pull out the taxonomy of the top hit, the top hit’s accession number, and the percent identity of the query to the top hit. We used both of these taxonomies to validate ASV identity. For further data analysis and quality control, we used R version 4.03 (66). We removed contaminants using our blank controls and the R-package decontam (ver 1.10.0) (67). Next, we exported the feature table from QIIME2, used decontam with the conservative threshold of 0.5 to identify contaminants, and then filtered the 19 identified contaminants from the feature table. We additionally removed any ASVs identified as mitochondria or chloroplasts. To normalize the number of sequences per library, we ran alpha-rarefaction in QIIME2 and selected 7,800 reads per sample to retain most samples while still capturing the majority of the diversity of the samples. We exported this rarefied feature table (with chicken bait and wasp samples removed) and used the R vegan package (ver 2.5–7) (68) for further analysis.

To investigate beta diversity, we first calculated Bray-Curtis dissimilarities and nonmetric multidimensional scaling using metamds. To visualize the resulting ordination, we used ordiellipse. We then tested for differences between bee gut microbiomes based on diet (obligate, facultative, or absent necrophagy) and species using separate Adonis models. In both models we accounted for potential nonindependence of baits by adding location as a block. For the bee species models, we removed species with fewer than three individuals, retaining 10 species. We used the vegan betadisper function to test for homogeneity of multivariate dispersions using location as a block.

To investigate patterns in alpha diversity, we ran linear mixed effects models (LMMs) using the lme4 package in R (ver. 1.1.27.1) (69). We tested two models that included the number of ASVs in bees as response variables. The first model included diet (obligate, facultative, or absent necrophagy) as the predictor variable, as well as bee species and location as the random effects. We could not compare diet and species within a single model because they are colinear and so instead evaluated a second model that included ASV as the response variable, with species and the collection substrate (bait, flower, or other) as predictor variables and location as the random effect. The data followed a normal distribution and were well described by the LMMs (70).

To determine statistical significance, we conducted likelihood ratio tests in which the variable of interest was removed and compared to an otherwise equivalent model. We evaluated pairwise comparisons among species via *post hoc* tests (Tukey’s honestly significant difference [HSD]; R package emmeans ver 1.6.2.1 [71]). Finally, we used ancom in QIIME2 to test for ASVs that were differentially abundant between the microbiomes of bees across dietary lifestyles (72).

### Data availability.

Raw sequencing data are available on the NCBI Sequence Read Archive under BioProject PRJNA749807 and Biosample accession numbers SAMN20418664 to SAMN20418836. Additional data and R code are available at https://github.com/llf44/Vulture_bee_microbiome.
